# Learning from a multi-agency trauma-informed care training initiative supporting people experiencing homelessness in rural and coastal areas: a qualitative study

**DOI:** 10.1186/s12913-025-13371-8

**Published:** 2025-09-02

**Authors:** Steven A. Thirkle, Deepti A. John, Emma A. Adams, Jill Harland, Eileen Kaner, Sheena E. Ramsay

**Affiliations:** 1 Population Health Sciences Institute, Newcastle University, The Baddiley-Clark Building, Richardson Road Newcastle upon Tyne, Newcastle, NE2 4AX UK; 2 Northumbria Healthcare NHS Foundation Trust, Hexham General Hospital, Corbridge Road, Hexham, NE46 1QJ UK

**Keywords:** Trauma-informed care, Trauma, Homelessness, Rural and coastal, Multi-agency training

## Abstract

**Background:**

People experiencing homelessness have often faced both historical and ongoing trauma, which can be compounded by their interactions with different support services. Trauma-informed care aims to meet the complex needs of people who have experienced trauma and prevent re-traumatisation during service interactions. In rural and coastal areas, where services are often geographically dispersed, multi-agency approaches are particularly crucial for ensuring continuous, coordinated support. This study examines the learning from implementing a multi-agency trauma-informed training pilot for providers supporting people experiencing homelessness.

**Methods:**

A qualitative study was undertaken following a trauma-informed care training pilot delivered to health and social care providers in Northumberland and North Tyneside, two geographically diverse rural and coastal areas in North East England. Those who attended the training were invited to take part in follow-up semi-structured interviews, thirteen out of 16 attendees participated, representing healthcare, emergency care, housing, voluntary sector, and social care services. Interviews explored how training influenced trauma-informed practice and cross-service collaboration.

**Results:**

Interviews highlighted the value that participants of the training found in bringing different services together for undertaking trauma-informed training, and the opportunity for shared learning amongst those who provide care for people with multiple needs and interacting with different services. Three key themes were identified from the thematic analysis: (1) training cultivated awareness, sensitivity and compassion in communication across services (2) the need to foster support and trauma sensitivity through multi-agency collaboration and wider-system engagement and (3) building organisational and individual resilience through shared learning and coordinated supportive practices.

**Conclusions:**

Multi-agency trauma-informed training helps ensure consistent approaches across geographically dispersed services supporting people experiencing homelessness. When staff from different organisations train together, it builds a shared understanding of trauma, encourages collaborative working, and supports staff wellbeing. This integrated approach is particularly valuable in rural and coastal areas where service coordination can be challenging due to geographical dispersion and resource constraints.

**Supplementary Information:**

The online version contains supplementary material available at 10.1186/s12913-025-13371-8.

## Introduction

Homelessness is a significant public health concern that is increasing globally. The United Nations estimates that 1.6 billion people live in inadequate housing conditions [1]. Economic challenges, housing shortages, systemic inequalities, and inadequate support systems interact with individual circumstances and experiences to contribute to homelessness [1–3]. In 2023, similar factors contributed to over 300,000 households across Great Britain experiencing homelessness. In rural and coastal areas of the UK, homelessness is becoming increasingly prevalent, often manifesting in less visual forms such as sofa surfing (staying with friends or family on sofas) and temporary accommodation [2, 4, 5].

People experiencing homelessness face high levels of physical and mental health needs which are exacerbated by other issues such as poverty, unemployment, lack of education and, physical and sexual abuse [6]. The episodic nature of some experiences of homelessness and the challenges faced when engaging with traditional health and social care services often leave physical and mental health needs unaddressed [7]. The intrinsic connection between physical health, mental health, and social circumstances necessitates a holistic approach to care delivery [6, 7]. Therefore, multi-agency collaboration (i.e. different services, including health, housing, social care, welfare) is essential to effectively address immediate health needs and underlying social determinants [8, 9]. Despite the recognised need for coordinated support to address their needs, access to appropriate services for people experiencing homelessness remains limited and available services can be siloed and fragmented [10, 11].

In addition to multiple health and social challenges, people who experience homelessness are also likely to have experienced psychological trauma [12]. Trauma occurs ‘when we experience very stressful, frightening or distressing events that are difficult to cope with or out of our control’ [13]. More specifically, trauma results from an event, series of events, or set of circumstances that is experienced by an individual as physically or emotionally harmful or threatening, having lasting adverse effects on the individual’s functioning and physical, social, emotional or spiritual well-being [14]. People experiencing homelessness frequently encounter complex trauma, repeated exposure to multiple traumatic events often beginning in childhood and continuing through adulthood [15, 16]. This includes interpersonal violence, sexual abuse, neglect, and institutional trauma from repeated interactions with fragmented service systems. Complex trauma is particularly dangerous as it disrupts fundamental capacities for self-regulation, attachment, and meaning-making [17]. Systemic barriers including discriminatory practices, conditional service provision, and repeated assessment processes can compound existing trauma and create new traumatic experiences, perpetuating cycles of instability and service disengagement [18]. The homelessness experience itself is often traumatic [16, 19], as it is a circumstance that can often be physically and emotionally harmful and sometimes even life-threatening [14]. The precarious nature of homelessness, characterised by physical danger, emotional turmoil, and systemic barriers, further compounds the trauma experienced by this vulnerable population [20]. The institutional circuitry that people experiencing homelessness navigate, including fragmented services, repeated assessments, conditional support, and power imbalances within service systems, can itself be traumatising and exacerbate existing trauma.

In this context, trauma-informed care offers a promising framework, addressing the unique needs of the person experiencing homelessness and supporting service providers within their organisational settings [21]. Trauma-informed care is a strengths-based framework that recognises the pervasive impact of trauma and seeks to create environments that prioritise safety, trust, and empowerment [22]. Despite this, trauma-informed, as a term has faced criticism for its lack of universal definition, clarity on measurable outcomes, and evidence of effectiveness [23–25]. Where organisations attempt to implement trauma-informed care, they can face additional challenges including resource intensiveness, difficulties achieving implementation fidelity, and concerns about superficial adoption without meaningful systemic change [21, 26]. These implementation barriers can be particularly pronounced in rural and coastal areas where geographical dispersion and limited organisational capacity create additional challenges for sustained trauma-informed practice [27, 28].

By integrating trauma-informed principles into service delivery, organisations can better meet the needs of people experiencing homelessness, whilst also addressing the underlying trauma that perpetuates cycles of instability [18, 21, 29]. Through a multi-agency trauma-informed approach, organisations can provide coordinated care across service pathways, fostering resilience and continuity for people experiencing homelessness [9, 18, 21]. Such alignment of practice across organisations can help prevent re-traumatisation and create a more cohesive support network for vulnerable individuals navigating multiple services [9].

Multi-agency trauma-informed care training in rural and coastal settings offers unique advantages for addressing these challenges [9, 21]. When staff from different organisations train together, it helps break down silos between services and ensures consistent trauma-informed care across the entire system of care [18, 29]. This shared training approach is particularly valuable in rural and coastal areas where services are geographically dispersed [27, 28, 30], as it creates opportunities for providers to build relationships, develop shared understanding, and establish coordinated care pathways. Such collaborative training can help ensure that people experiencing homelessness receive consistent trauma-informed support regardless of which service they access [18, 21].

Despite these potential advantages, research examining trauma-informed care training presents mixed findings. Purtle’s systematic review of trauma-informed organisational interventions found modest improvements in staff knowledge and attitudes immediately post-training, though evidence for sustained practice changes remained limited [31]. Specific evaluation of trauma-informed care training for homelessness services demonstrated positive participant feedback but highlighted implementation challenges related to organisational capacity and resource constraints [26]. Existing research has often evaluated trauma-informed care training using quantitative methods [26, 31] but there is limited qualitative evidence on ways to integrate trauma-informed care into services that support people experiencing homelessness. In this study, we aimed to understand the learnings from a multi-agency trauma-informed training pilot amongst staff from different organisations across the health and care system in rural coastal areas in North East England. The objective of the study was to identify barriers and facilitators to adopting trauma-informed care in multi-agency settings. The findings aim to inform strategies for implementing trauma-informed practices across geographically dispersed support systems.

## Methods

A pragmatic paradigm supported an action-oriented framework for this research [32], where we sought to explore providers’ experiences and perspectives on adopting trauma-informed care across different services and organisational settings and general reflections following attendance at a trauma-informed care training pilot. A qualitative approach was chosen as it enabled understanding of learnings or outcomes from the training that were not easily measured and allowed for a more nuanced understanding of the impacts on their roles and organisations [33].

### Study setting

The study is based in Northumberland and North Tyneside, which are geographically diverse areas in North East England, distributed across almost 2000 square miles. Although both areas have predominantly white British populations (over 95% of residents), these areas have distinct demographic characteristics. Northumberland, primarily a rural county with approximately 325,000 residents, has a higher proportion of older individuals. North Tyneside, comprising rural, coastal, and suburban areas, has a smaller population of approximately 210,000 with a younger median age [34].

### Training intervention

The training was developed by Changing Lives (a UK-based charitable organisation specialising in supporting vulnerable populations) and focused on adopting trauma-informed care to support people experiencing homelessness. The training aimed to foster a comprehensive understanding of trauma-informed care based on principles of understanding, recognising, and responding to the effects of all types of trauma. It was seeking to emphasise physical, psychological, and emotional safety for both service users and service providers and help people who have experienced trauma rebuild a sense of control and empowerment. Traditionally, the training had been run for individual organisations, but in this context the training was customised to enable a cohort of attendees from different services/sectors that often work individually and together to support the care and needs of people experiencing homelessness.

Representatives from multiple organisations were invited to participate and attendees were a self-selecting sample, including statutory services, healthcare providers, voluntary sector organisations, emergency services, and housing support services. Sixteen staff members attended the full day training program, which covered both theoretical frameworks and practical challenges of applying trauma-informed methods in their daily work.

### Sampling and data collection

All participants who attended the trauma-informed care training session were invited to participate in follow-up interviews 10 weeks after the training. Thirteen of the 16 staff who attended the trauma-informed care training were recruited to participate in the study. Participants received detailed study information and written, or audio-recorded consent was obtained before participating. To maintain independence from the training, the lead author (experienced in qualitative research) conducted all the semi-structured interviews (a copy of the topic guide can be found in the supplementary material). Participants were explicitly assured that responses would remain confidential and would not be shared with training providers or their employers. Interview questions emphasised both positive and challenging aspects of implementation, encouraging candid reflection on difficulties encountered. Most interviews were conducted virtually with a few taking place at service premises. The National Health Service (NHS) Health Research Authority (HRA): National Research Ethics Service Committee in West Midlands Edgbaston provided ethical approval for this study, in accordance with the Declaration of Helsinki (Research Ethics Committee reference: 22/WM/0099). Participants provided written informed consent in accordance with the Declaration of Helsinki.

### Analysis

Interviews were digitally recorded, transcribed verbatim, and checked for accuracy. Processed transcripts were imported into NVivo V. 14 to support data management and analysis [35]. For anonymity purposes, all identifiers (including participant’s and non-participant’s characteristics such as name, gender and age) were removed to maintain anonymity.

The interview transcripts and field notes were analysed thematically [36], supported by the framework method [37]. Initial deductive codes were based on the research questions and literature review. The data’s richness and diversity allowed for a wide exploration of content, leading to a nuanced understanding of the themes. The research team familiarised themselves with the interviews through multiple readings and listening sessions, ensuring deep engagement with the data. Each interview was coded line-by-line using the three main topic questions as parent codes: impact on practice, organisational integration, and barriers and facilitators to implementation. Relevant child codes were sorted beneath these parent codes. Patterns and initial themes were identified during this coding process. Final themes were developed through collective analysis of the codes, initial themes, supporting quotes, and explanatory notes. The first and second authors undertook the coding of the transcripts, and other authors verified the grouping of codes and final themes.

## Results

Of the thirteen staff who took part in the interviews ten were females and three males, with ages ranging from 18 to 64 years. Participants represented a range of professional backgrounds including paramedics, health trainers, public health workers, and managers from housing and support services. The participants held various positions including both frontline staff and senior management roles, providing diverse perspectives on trauma-informed care implementation. Participants had an average of 5 years experience in their current roles, indicating a relatively experienced sample with sustained engagement in their respective services. Length of interviews were on average 28 min in duration.

Three themes were developed from the data analysis: (1) Multi-agency Trauma-Informed Care Training Cultivates Awareness, Sensitivity, and Compassion (2) Need to Foster Support and Trauma Sensitivity Through Collaboration Across Services and the Wider System and (3) Building Organisational/Individual Resilience and the Need to Encourage Supportive Practices.

### Theme 1: Multi-agency trauma-informed care training cultivates awareness, sensitivity, and compassion in communication

The training sessions emphasised the significance of using compassionate language and encouraged real-time reflection which was found to be valued by participants across different service settings, especially when supporting people experiencing homelessness.

*“One of the things that resonated the most was*,* like*,* not thinking or saying to somebody*,* like*,* ‘What’s wrong with you?’ but saying*,* ‘What’s happened to you?‘” – P3*.

Another aspect found to be useful in changing practice was related to the importance of maintaining expectations and boundaries with their service users through open and clear communication, regardless of their organisational role.

*“Because we are still working with those vulnerable people*,* so it’s maybe changed it more in the way of just being more aware of how to maybe communicate with them*,* but also take how they might respond to things to you. The main thing that it has kept in my head is that transparency side of things. I think that’s the main thing. It’s letting them know what you’re doing*,* why you’re doing it*,* and what the next steps are. I think that’s the main thing that has influenced my practice.” – P5*.

However, participants also acknowledged challenges with trauma-informed terminology and concepts.

*“I just think that bit there*,* to be honest with you*,* the bit that says ‘whole-system transformation*,* advocate for system-wide adoption of trauma-‘*,* it’s a bit of a mouthful*,* isn’t it? I just think I’ve switched off after…. Do you know what I mean? And then I don’t quite understand what it’s talking about*,* to be honest with you.” – P7*,* 8*,* 9*,* and 10* While training enhanced trauma-informed communication skills, participants acknowledged ongoing challenges with complex terminology and practical application in daily practice. *“I had a gentleman in the hospital*,* he was sleeping in a tent at the time. I’d spoken to him on the phone and I’d meant to say… I should have said*,* ‘Have you been discharged?’ but I said*,* ‘Are you home yet?’ You know*,* and he realised straight away… I mean*,* have you been discharged?’ He could have just been like*,* ‘No*,* I haven’t got a home*,* have I?’ Just being really quite sensitive about your language. I realised straight away what I’d said.” – P4*.

### Theme 2: Need to foster support and trauma sensitivity through collaboration across services and the wider system

Participants felt the training enabled them to recognise the importance of comprehending trauma’s impact, particularly in the context of homelessness and integrating trauma-informed practices into daily operations across different service settings.

*“I think the more that people understand it*,* and the benefits*,* the easier it will be for people to even do some of it themselves and look at some of the links and try and understand some of it. Even the basic thing*,* like I said before. Even the definition of understanding what trauma is.” – P11*.

The need for more accessible resources in rural and coastal areas, along with practical tools (e.g. knowing how to respond, where to seek help), was highlighted as crucial for effective implementation of trauma-informed practices. Hands-on experiences, such as training and shadowing across different services, solidified the understanding and application of trauma-sensitive approaches, benefiting both themselves and the people they support.

*“I guess it’s creating those strategies that team members then can quickly use*,* whether that is*,* ‘Actually*,* we have a safe*,* like a quiet room. You can go and take that person for a conversation there*,*’ and that’s something that we dedicate and we have in our service*,* or you can say you need 10 more minutes to make someone a cup of tea and that’s absolutely acceptable. I think it’s having those things that people can just pick up and use in their day-to-day tools. We all need those cognitive jumps to make work easy when you’ve got a lot going on in a clinical area.” – P12*.

*“So*,* a brief shadow of something*,* to shadow something and then you understand. So*,* it could even be doing a bit of training and then being able to shadow that*,* just to actually understand what that looks like in principle.” – P5*.

Despite recognising collaboration benefits, participants highlighted practical implementation challenges.

*‘When you’re in an A&E clinical setting*,* how do you know that straightaway about someone? How do you do what’s most appropriate for that person? I think it’s almost opening a can of worms for some services*,* I think*,* in terms of how they apply that and what they do next.’ – P12*.

Concerns about system-wide buy-in were also evident.

*‘If the decision makers don’t prioritise this then there’s not going to be that dissemination’ – P12*.

Though multi-agency collaboration was valued, participants identified significant organisational and systemic barriers to sustained implementation. *“And I think that probably comes from everybody doing it. So I do it*,* people do it to me. I think people would be certainly open and frank with me without worrying*,* ‘Oh*,* he’s a director*,* you can’t do this.’ But equally I don’t pretend that I haven’t got any problems. If there’s stuff going on*,* I’ll come in and say*,* ‘Oh God*,* this is happening.’” – P10*.

### Theme 3: Building organisational/individual resilience and the need to encourage supportive practices

Participants acknowledged the significance of leveraging ongoing learning initiatives and adopting a trauma-informed approach to promote resilience and enhance service delivery effectiveness across the system.

*“And well-being*,* even for yourself. So*,* even some of my colleagues who were on the course*,* one of the things I would say that we came away talking about is how much we probably don’t even consider our own well-being? And how can you help other people if you are burnt out? If you’re not well*,* if you’re not looking after yourself*,* and doing things?” – P11*.

Discussions focused on the importance of building resilience within staff members, understanding triggers, and implementing tools and techniques to navigate challenges when supporting people experiencing homelessness effectively (for example understanding triggers in service users, knowing where to find help and how to respond).

*“How can we build resilience in staffing*,* in order to deal in process with lots of clients?’ and their presentation*,* in terms of their vulnerability. So*,* you know*,* tools and techniques*,* I guess understanding the triggers in ourselves. You know*,* therefore*,* what we’re going to take from that.” – P2*.

There was a general recognition by the participants about the dispersed nature of services, especially in the rural and coastal settings, which made training for trauma-informed practices and wider collaboration challenging.

*“I think the challenge that public services have in terms of being stretched in a number of different ways and a lack of capacity for learning. But*,* also*,* lack of capacity around burnout and staff being a bit frustrated by some of the nature of some of the calls.”- P6*.

Participants acknowledged the significant time investment required for trauma-informed care, recognising that while these approaches align with their values, the additional time demands create ongoing tensions with operational pressures and may not always yield expected outcomes. *“When it comes to the work that we’re talking about here*,* I completely 100% agree that it’s what we should be doing*,* but naturally*,* it’s going to take a little bit longer to do*,* because of that conversation and getting people on side and getting people to share with you and all that kind of thing*,* which is all completely the right thing to do. But*,* naturally*,* it takes time and it’s just a complete opposite of what Operational Management’s focus is. So*,* it’s always going to be up against that. Like I say*,* I completely agree that this is the right thing to do*,* and this is the right way to approach people… but*,* because it takes time… something like this that then is going to increase time*,* like I say*,* is at odds with that.” – P3*.

## Discussion

The findings of this study enabled us to understand the learnings from how trauma-informed care training could support service provision for people experiencing homelessness in rural coastal areas of North East England. The training cultivated awareness and compassion among providers while highlighting the importance of multi-agency collaboration and organisational resilience in implementing trauma-informed care effectively.

This study’s findings contribute to the existing literature on trauma-informed care for professionals working with people experiencing homelessness by emphasising the importance of practical, hands-on training experiences and the need for ongoing support and resources to implement trauma-informed care principles effectively [18, 21, 29]. These findings align with previous research that emphasises the need for trauma-informed care in service provision for people experiencing homelessness [12]. The shift in perspective from “What’s wrong with you?” to “What’s happened to you?” [16, 22], echoes the core principles of trauma-informed care as outlined by SAMHSA [14] and demonstrates how these principles can be integrated into daily practice. The importance of compassionate language and transparent communication identified in this study supports previous findings on the effectiveness of trauma-informed care in services supporting people experiencing homelessness [18, 21, 29].

The emphasis on multi-agency collaboration reflects the complex, multi-factorial nature of homelessness and trauma, which requires coordinated responses across health, housing, social care, and emergency services to address interconnected needs that no single agency can meet independently [19, 38]. Our findings extend this understanding by highlighting implementation challenges specific to rural and coastal areas, a context that has received limited attention in previous research [18, 21, 29]. These implementation challenges align with recent findings on service coordination in the region [11]. The geographical spread of services often results in isolation and fragmentation, complicating efforts for inter-agency collaboration crucial to trauma-informed care [28]. Resource constraints, a common issue in rural and coastal services [27], further complicate implementation efforts. While participants valued the opportunity to train together, they identified ongoing challenges related to service capacity and geographical dispersion that could impact implementation of trauma-informed care.

The focus on building organisational and individual resilience aligns with challenges identified in previous studies, such as limited funding, understaffing, and overwhelming workloads [27, 28]. This tension emphasises the need for tailored strategies that can effectively support staff within the unique operational context of rural and coastal services, where staff often juggle multiple responsibilities with limited support.

### Strengths and limitations

A key strength of this study is its qualitative approach, which builds on existing quantitative evidence and allows for an in-depth exploration of service providers’ experiences following trauma-informed care training. The inclusion of diverse service sectors provides a holistic view of implementation challenges across the care continuum in rural and coastal areas. However, the study has several limitations. It focuses on service providers’ perspectives following a training session, which may not capture long-term impacts or the views of people experiencing homelessness themselves. Additionally, self-selection of participants in the training and subsequent interviews may have resulted in a sample biased towards those with a positive view of trauma-informed care. It is possible that participants in the study were experienced staff and a motivated group, which could have influenced their perspectives on trauma-informed care implementation. The positive participant responses may reflect social desirability bias, despite efforts to encourage candid feedback. Participants may have felt reluctant to express criticism of the training or implementation challenges. Future studies could benefit from incorporating more diverse viewpoints, perhaps including participants who were unfamiliar with trauma-informed care or those who are not in support of it and service users.

### Implications and conclusions

This study highlights several key features for developing trauma-informed care approaches in services supporting people experiencing homelessness, particularly in rural and coastal settings where homelessness may be less visible but equally impactful [2, 5]. By fostering awareness, promoting multi-agency collaboration, and building resilience, trauma-informed care offers a promising framework for addressing complex needs in services supporting people experiencing homelessness. The benefits likely extend beyond the immediate target group, potentially improving outcomes for the broader community and addressing wider health inequalities. However, realising this potential requires ongoing commitment, resources, and support at both organisational and policy levels, particularly in addressing the unique challenges posed by rural and coastal contexts. Table 1 presents general recommendations for decision-makers looking to implement multi-agency trauma-informed care training in rural and coastal areas supporting people experiencing homelessness.

Future research is needed to evaluate the long-term impacts of training on service delivery and outcomes for people experiencing homelessness, particularly in rural and coastal settings. Research to quantify the return on investment of trauma-informed training practices would also be beneficial to understand financial implications. Additionally, investigating the perspectives of people experiencing homelessness on trauma-informed care approaches would provide useful understanding for improving service provision.

**Table 1:** Recommendations for implementing multi-agency trauma-informed care training in rural and coastal services supporting people experiencing homelessness

Target area for recommendations	Specific recommendations
Multi-agency implementation	• Establish standardised trauma-informed training across services • Create shared language and communication approaches • Develop sustainable cross-agency learning networks • Build mechanisms for ongoing collaboration
Rural and coastal service delivery	• Design flexible delivery methods for geographically dispersed areas • Provide accessible resources and digital solutions • Maintain service consistency across isolated locations • Create pathways for remote support and communication
Organisational development	• Embed trauma-informed principles in policies and practice • Allocate protected time for training and reflection • Implement practical tools (or tips and ways of working) to apply to daily work • Establish supervision and peer support structures
Staff capacity building	• Enhance understanding of trauma triggers and responses • Develop trauma-sensitive communication skills • Foster resilience through well-being support • Create opportunities for shared learning

## Supplementary Information

Supplementary Material 1.

## Data Availability

The data that support the findings of this study are not openly available due to reasons of sensitivity. The information collected contains potentially identifiable information, and releasing the raw data could compromise participant confidentiality. However, summaries of the data are available from the corresponding author upon reasonable request.
