# Supplementary material for: Learning from a multi-agency trauma-informed care training initiative supporting people experiencing homelessness in rural and coastal areas: a qualitative study
Source: BMC Health Serv Res. 2025 Sep 2;25:1175. doi: 10.1186/s12913-025-13371-8 (PMC12403314; doi:10.1186/s12913-025-13371-8)
Supplement: Supplementary file 1 — Supplementary Material 1. [file 12913_2025_13371_MOESM1_ESM.docx]

**Trauma-Informed Care Training Pilot for Supporting Services in Northumberland and North Tyneside: Interview Topic Guide**

**Aim of pilot study/ evaluation**

The aim of this pilot study is to evaluate the implementation and learning from trauma-informed training delivered to services supporting people experiencing homelessness.

**Objectives of pilot study/ evaluation**

1. Capture change in participants’ understanding, knowledge and awareness of trauma-informed care
2. Assess participants’ confidence and capacity in applying trauma-informed care in their practice
3. Identify strategies to take the learning forward into practice
4. Identify barriers to the long-term adoption and implementation of trauma-informed care in their organisations
5. Understand the perceived impact of the training on participants' service delivery
6. Identify elements of trauma-informed approaches for a toolkit that is tailored and used by services/ organisations

**Topic-Guide**

1. **Do you have any questions?**

I’d like to go through a few questions just to make sure that you are happy to take part in the study, and how we collect your information for research.

1. First, do you agree that you understood what this study is about and you’ve had the chance to ask us questions?
2. Do you agree that your taking part is voluntary and you are free to withdraw any time without giving a reason?
3. Our chat is being audio recorded. The recordings will be stored securely without your name and any other personal details and will only be used for research purposes only for this study and further research. We will have your name and contact details only for contacting you for this study. Is that ok with you?
4. The information we talk about today (or data) will be used as part of our research and will later be published as a report, without any names or personal details. Are you happy with that?
5. So, are you happy to take part in this research study?

**Objective 5: Understand the perceived impact of the training on participants' service delivery**

**Question 1)**

1) How has this training changed the way you work or respond differently to service users who experience homelessness?

**Prompts**

- Is there anything about the training that was not useful or could be delivered differently?
- Was there anything missing from the training to help you support TIC?
- In what ways do you believe the training has improved your ability to provide services to people who have experienced homelessness and trauma?
- Have you noticed any changes in your interactions with service users or the overall outcomes of their services because of the training?
- What specific elements or concepts from the training do you feel have had the most impact on your service delivery?
- Have you found anything difficult in applying the training to your role?

**Objective 3: Identify strategies to take the learning forward into practice**

**Question** **2)**

2) What are some ways that you think what you have learned can be applied more widely in your organisation?

**Prompts**

- Who else would benefit from attending TIC training
- How can staff be encouraged and supported to apply their learning from the training in their day-to-day work?
- What resources or tools would benefit staff (and services) in their implementation of the learning from this training into their service?
- Are there opportunities in your service for ongoing education and training to deepen the understanding and application of trauma-informed care?
- What else is needed to create a trauma informed safe environment?
- How do you think senior management/leaders can support the integration of trauma-informed practices into the organisation?
- Does TIC align with any existing approaches/practices used within your role/ service

**Objective 4: Identify barriers to the long-term adoption and implementation of trauma-informed care in their organisations**

**Question 3)**

3) What barriers do you think your service will face – or what might help – when trying to embed, adopt and implement trauma-informed care in the long term?

Prompts

- What do you think would be helpful/ or what is needed to create a trauma informed environment/culture within your organisation?
- Are there any organisational or systemic factors that may prevent sustaining trauma-informed care?
- What are the challenges or barriers? [probe, if needed on below resource, administrative]
- What might help adopt a trauma-informed approach? What would your organisation need?
- How can resistance or reluctance from staff members (or services) be addressed and overcome?
- What potential resource-related barriers may develop when implementing and maintaining trauma-informed care?
- Are there any legal or legislative barriers that services will need to consider when implementing trauma-informed care?

**Objective 6: Identify elements of trauma-informed approaches for a toolkit that is tailored and used by services/organisations**

**Question 4)**

4) We have drafted a resource/toolkit to help services/ organisations that support people experiencing homelessness become more trauma-informed. Please take a look.

- Does it look useful?
- Does it have the key components?
- Do you think it will be helpful going forward?
- Do you think that it has the potential to be easily tailored for services and organisations?
- Who would use the toolkit (target audience?)
